# Comparison of root resorption severity after fixed and invisible orthodontic treatment in patients with four first premolar extractions: A retrospective study

**DOI:** 10.1097/MD.0000000000048605

**Published:** 2026-05-01

**Authors:** Li Su, Qichao Kang, Xu Zhang, Dan Li, Chen Luo, Zhe Tang, Jingwen Wang, Norma Ab Rahman

**Affiliations:** a Orthodontic Unit, School of Dental Sciences, Universiti Sains Malaysia, Kubang Kerian, Kelantan, Malaysia; b Department of Stomatology, Beijing Xuanwu Tcm Hospital, Beijing, China; c Department of Stomatology, Beijing Luhe Hospital, Capital Medical University, Beijing, China; d Center for Microscope Enhanced Dentistry, School of Stomatology, Beijing Stomatological Hospital, Capital Medical University, Beijing, China; e Department of Orthodontics, School of Stomatology, Beijing Daxing Xingye Stomatology Hospital, Beijing, China; f Department of Orthodontics, Changzhi People’s Hospital, Changzhi Medical College, Changzhi, Shanxi, China; g Department of Stomatology, Tianjin First Central Hospital, Tianjin, China.

**Keywords:** cephalometric analysis, clear aligners, fixed appliances, orthodontic extraction, root resorption

## Abstract

Root resorption is a common and unavoidable complication of orthodontic treatment, with limited research on its pattern in extraction cases using clear aligners. This study compared the severity of root resorption between fixed appliances and clear aligners in patients who underwent 4 first premolar extractions. A retrospective analysis was conducted on 28 patients with Angle Class I and II malocclusion treated with fixed appliances (n = 14) or clear aligners (n = 14) from 2019 to 2025. The mean treatment duration was 37.93 ± 13.65 months. Root resorption was assessed for both groups involving 488 teeth for right and left first molar and anterior teeth until canine for both arches excluding first and second premolar using the relative root-to-crown ratio on panoramic radiographs and tooth movement was measured using cephalometric superimposition. Chi-square and Mann–Whitney *U* tests were used to evaluate group differences. The proportion of teeth with no root resorption was significantly higher in the clear aligner group (52.67%, 236 teeth; χ^2^ = 11.253, *P* = .010) compared fixed appliance group (42.86%, 192 teeth). For upper anterior teeth, the fixed appliance group had a notably higher rate of moderate-to-severe resorption (40.91%) compared to the clear aligner group (17.14%; χ^2^ = 33.651, *P* < .001). For upper first molars and lower first molars, the clear aligner group also showed higher proportions of teeth without resorption (upper first molars: 28 vs 20 teeth, χ^2^ = 11.333, *P* = .010; lower first molars: 36 vs 16 teeth, χ^2^ = 15.692, *P* = .001). In terms of tooth movement, only the vertical (*Y*-axis) movement of lower anterior teeth differed significantly between groups: 0.8814 ± 1.01909 mm (fixed appliances, 95% CI: 0.06107–1.82393) versus 1.1314 ± 0.70874 mm (clear aligners, 95% CI: 0.47595–1.78690; *P* = .017), with no significant differences in other directions (all *P* > .05). Fixed appliances may increase the risk of moderate-to-severe root resorption in anterior teeth due to continuous heavy forces, while clear aligners reduce severe resorption but require caution during vertical movements.

## 1. Introduction

Root resorption is one of the most common complications of orthodontic treatment and is unpredictable and inevitable.^[1,2]^ As a new orthodontic technique, the principles and clinical applications of clear aligners significantly differ from those of fixed appliances. Previous studies have investigated the patterns and risk factors of root resorption during clear aligner treatment.

However, with the development of clear aligner technology, earlier research may not fully reflect the current clinical situation of root resorption in patients undergoing extraction. Extraction cases are uniquely challenging because they involve significant anterior retraction and posterior movements that exert greater mechanical stress on root surfaces and periodontal ligaments (PDLs) compared to non-extraction treatments. The mechanical difference in extraction cases stems from the need to close large extraction spaces: fixed appliances rely on arch wire tension to pull anterior teeth posteriorly, which can create concentrated stress at the root apex. Clear aligners, by contrast, use tray-mediated tooth rotation and translation, which may distribute stress more evenly across the root surface but how this translates to resorption risk in extraction cases has not been fully elucidated, especially with recent advancements in clear aligner material and attachment design that have improved force precision.^[3]^

This study aimed to analyze root resorption patterns in patients with 4 first premolar extractions using panoramic radiographs, and to compare the influencing factors between fixed and clear aligners via cephalometric measurements. The study aimed to compare the severity of root resorption between fixed appliances and clear aligners in patients with Angle Class I and II malocclusion who underwent 4 premolar extractions. The hypothesis of this study was that clear aligners would result in a lower prevalence of moderate-to-severe root resorption compared to fixed appliances, particularly in anterior teeth (which undergo the most extensive movement in extraction cases). Additionally, we hypothesized that tooth movement parameters would differ between the 2 appliance groups and correlate with resorption severity. By analyzing root resorption via relative root-to-crown ratio (rRCR) on panoramic radiographs and quantifying tooth movement through cephalometric superimposition, this study seeks to provide evidence-based guidance for treatment planning in extraction cases ultimately helping clinicians balance treatment efficacy with root health.

## 2. Materials and methods

### 2.1. Study design and ethical approval

This retrospective observational study adhered to the Strengthening the Reporting of Observational Studies in Epidemiology Statement to ensure transparent and comprehensive reporting of methods and results. Ethical approval was obtained from the Ethics Committee of Beijing Xuanwu Tcm Hospital (Approval No. 2024-002-02) prior to the initiation of data collection. All procedures were conducted in accordance with the principles of the Declaration of Helsinki. Informed written consent was obtained from all participants (or their legal guardians for minors) before treatment, including consent for the use of their clinical and radiological data for research purposes.

### 2.2. Inclusion and exclusion criteria

Inclusion criteria were: underwent orthodontic treatment with either fixed appliances (stainless steel brackets, 0.022-in slot) or clear aligners (Invisalign, Align Technology); had complete pretreatment and posttreatment imaging examinations include curved sections and cephalometric measurements; were with permanent dentition; had no history of previous orthodontic treatment, endodontic therapy, or traumatic dental injuries; and had no systemic diseases affecting bone metabolism.

Exclusion criteria included: incomplete clinical records; poor-quality images with artifacts affecting root morphology visualization; and loss to follow-up before treatment completion.

### 2.3. Sample size calculation

Sample size calculation was performed using G Power 3.1.9.7 software (Heinrich-Heine-Universität Düsseldorf, Germany) based on preliminary data from a pilot study and previous literature.^[4]^ The primary outcome was the difference in the proportion of teeth with moderate-to-severe root resorption between the 2 groups. Assuming a significance level (α) of 0.05, a power (1-β) of 0.80, a projected proportion of moderate-to-severe resorption of 35% in the fixed appliance group and 20% in the clear aligner group, and an attrition rate of 10%, the minimum required sample size was determined to be 12 patients per group. To account for potential confounding and improving statistical robustness, the final sample size was set to 14 patients per group 28 patients total, 448 teeth evaluated, which exceeded the minimum required size.

Twenty-eight patients (448 teeth) were included (6 males, 22 females; mean age: 17.07 ± 7.68 years; mean treatment duration: 37.93 ± 13.65 months).

### 2.4. Fixed appliance protocol

Fixed appliances consisted of Damon 1 self-ligating fixed brackets (Ormco) bonded to all teeth except third molars. The archwire sequence was standardized as follows: 0.014-in nickel-titanium (NiTi) → 0.016-in NiTi → 0.016 × 0.022-in NiTi → 0.018 × 0.025-in NiTi → 0.016 × 0.022-in stainless steel → 0.018 × 0.025-in stainless steel. Three clinicians participated in this clinical trial, and a unified standard was adopted.

### 2.5. Clear aligner protocol

Clear aligners (Invisalign, Align Technology) were designed using Invisalign Outcome Simulator software, with treatment planning overseen by a single certified Invisalign provider. Aligner mechanics and staging were standardized as follows:

Attachment design: Button attachments (dome-shaped, 3 mm diameter) bonded to upper canines and first molars to enhance anchorage and improve tooth movement precision; rectangular attachments (2 × 3 mm) on upper central incisors to control torque (+10° for central incisors, +8° for lateral incisors).

Staging: Each aligner set was designed to induce 0.25 to 0.3 mm of tooth movement per aligner, with a wear time of 22 hours per day and aligner changes every 7 days.

Refinements: A single refinement stage was implemented mid-treatment (after approximately 50% of planned movement) to correct any deviations from the treatment plan and optimize root position.

### 2.6. Imaging and measurement

Panoramic radiographs: Acquired using a Planmeca ProMax 3D Mid (Planmeca, Helsinki, Finland) with standardized settings: 70 kVp, 10 mA, exposure time 14.8 seconds. Patients were positioned with the Frankfort plane parallel to the floor and the mid-sagittal plane aligned with the machine’s central axis to minimize distortion.

Lateral cephalometric radiographs: Acquired using the same device with settings: 80 kVp, 12 mA, exposure time 1.8 seconds. Head position was standardized using a head holder to ensure consistent alignment of the Frankfort plane and mid-sagittal plane.

### 2.7. Root resorption assessment

**Figure 1.**
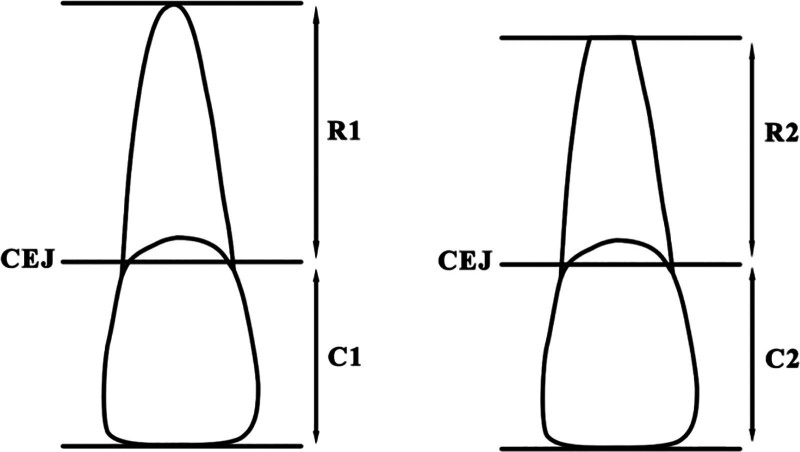
Root resorption measurement method (rRCR = (R2/C2)/(R1/C1), CEJ: cementoenamel junction).

Root resorption severity was evaluated using the Linge & Linge Root Resorption Grading System,^[5]^ a validated, widely used scale that quantifies resorption based on the rRCR. In panoramic radiographs, the long axis of the tooth was divided into the crown (C) and root (R) at the cemento-enamel junction. The pretreatment crown length was defined as C1, root length was defined as R1, posttreatment crown length was defined as C2, root length as R2. The rRCR was calculated as rRCR = (R2/C2) (R1/C1) × 100% (Fig. 1).

Root resorption was diagnosed if rRCR was < 100%. The specific grading criteria were as follows.

- No resorption: rRCR ≥ 100%.
- Mild (0–10%): 90% ≤ rRCR < 100%.
- Moderate (10–20%): 80% ≤ rRCR < 90%.
- Severe (>20%): rRCR < 80%.

To ensure measurement reliability, 20% of the samples (5 patients, 80 teeth) were randomly selected for reevaluation 2 weeks later. Intraclass correlation coefficient (ICC) was used to assess intra- and inter-examiner reliability, with ICC > 0.8 considered excellent agreement. The final ICC values for intra- and inter-examiner reliability were 0.92 and 0.87, respectively, confirming high measurement consistency.

### 2.8. Tooth movement measurement

- Superimposition Method: Cephalometric radiographs before and after treatment were structurally superimposed using the palatal plane for the upper anterior teeth and mandibular plane for the lower anterior teeth as a reference. The pretreatment apical position was transferred to the posttreatment image via the incisal edge point and dental long-axis superimposition to eliminate interference from changes in root length.
- Coordinate System Establishment: A two-dimensional coordinate system was established with the maxillary and mandibular planes as the reference planes and the pretreatment apical point as the origin. The displacement of the apical point in the vertical (*Y*-axis, extrusion/intrusion) and sagittal (*X*-axis, labial/palatal) directions before and after treatment was measured.

### 2.9. Reliability assessment

To evaluate intra-examiner reliability, the orthodontist repeated measurements for all 28 patients (448 teeth) 2 days after the initial measurement (this interval was chosen to minimize recall bias and is unrelated to treatment duration). ICCs were calculated for rRCR values, with ICC > 0.80 considered excellent reliability. The results showed an ICC of 0.92 for rRCR, confirming high intra-examiner consistency. Additionally, 10% of the sample (3 patients, 48 teeth) was randomly selected for assessment by a second independent orthodontist (with 8 years of experience) to evaluate inter-examiner reliability, yielding an ICC of 0.87 (excellent agreement).

### 2.10. Sensitivity analyses

Two prespecified sensitivity analyses were conducted to verify the robustness of the primary findings in the 28-patient cohort.

Using the alternative root resorption grading standard, 2 calibrated examiners (ICC = 0.91 for inter-examiner reliability) reassessed all teeth from the 28 patients, and inter-group comparisons were repeated to confirm that conclusions were not dependent on specific grading thresholds.

Reliability analysis results, detailed sensitivity analysis data, and raw measurement data for all 28 patients are available from the corresponding author upon reasonable request.

### 2.11. Statistical analysis

SPSS 27.0 was used for chi-square tests to compare the root resorption severity between the groups.

Mann–Whitney *U* tests were applied to non-normally distributed tooth movement data. All measurements were performed by a single trained orthodontist, with repeat measurements of 28 patients (448 teeth) after 20 days to control errors.

## 3. Results

A total of 28 patients (6 males, 22 females) were included in this study, with a mean age of 17.07 ± 7.68 years and a mean treatment duration of 37.93 ± 13.65 months. The participants were equally divided into 2 groups: the fixed appliance group (n = 14, involving 224 teeth) and the clear aligner group (n = 14, involving 224 teeth). There were no significant differences in age, gender distribution, or treatment duration between the 2 groups (all *P* > .05), indicating good comparability.

There was a statistically significant difference in the distribution of root resorption severity between the fixed appliance group and the clear aligner group (χ^2^ = 11.253, *P* = .010). Specifically, the proportion of teeth with no root resorption was significantly higher in the clear aligner group than in the fixed appliance group (52.67%, 236 teeth, 95% CI: 48.32%–57.02% vs 42.86%, 192 teeth, 95% CI: 38.57%–47.15%).

The fixed appliance group showed slightly higher proportions of moderate resorption (17.86%, 40 teeth, 95% CI: 14.01%–21.71%) and severe resorption (16.07%, 36 teeth, 95% CI: 12.32%–19.82%) compared with the clear aligner group (moderate: 11.61%, 26 teeth, 95% CI: 8.12%–15.10%; severe: 14.29%, 32 teeth, 95% CI: 10.68%–17.90%). The proportion of mild resorption was similar between the 2 groups (fixed appliance group: 23.21%, 52 teeth, 95% CI: 19.06%–27.36%; clear aligner group: 21.43%, 48 teeth, 95% CI: 17.34%–25.52%). Detailed data are presented in Table 1.

**Table 1:** Comparison of root resorption severity between the 2 appliance groups.

Root resorption severity	Fixed appliances	Proportion (%)	95% CI	Clear aligners	Proportion (%)	95% CI	χ^2^	*P*
No resorption	192 teeth	42.86	38.57%–47.15%	236 teeth	52.67	48.32%–57.02%	11.253	.010
Mild resorption	52 teeth	23.21	19.06%–27.36%	48 teeth	21.43	17.34%–25.52%	–	–
Moderate resorption	40 teeth	17.86	14.01%–21.71%	26 teeth	11.61	8.12%–15.10%	–	–
Severe resorption	36 teeth	16.07	12.32%–19.82%	32 teeth	14.29	10.68%–17.90%	–	–

A significant difference was observed in the distribution of root resorption of upper first molars between the 2 groups (χ^2^ = 11.333, *P* = .010). The number of upper first molars with no resorption was significantly higher in the clear aligner group than in the fixed appliance group (28 teeth, 95% CI: 19.85%–36.15% vs 20 teeth, 95% CI: 12.58%–27.42%).

The number of teeth with mild or moderate resorption was similar between the 2 groups (fixed appliance group: 12 teeth with mild resorption, 12 teeth with moderate resorption; clear aligner group: 4 teeth with mild resorption, 4 teeth with moderate resorption). There was a slight difference in the number of teeth with severe resorption (fixed appliance group: 12 teeth, 95% CI: 5.82%–18.18%; clear aligner group: 20 teeth, 95% CI: 12.58%–27.42%).

There was a statistically significant difference in root resorption distribution of lower first molars between the 2 groups (χ^2^ = 15.692, *P* = .001). The clear aligner group had a notably higher number of lower first molars with no resorption compared with the fixed appliance group (36 teeth, 95% CI: 27.42%–44.58% vs 16 teeth, 95% CI: 9.35%–22.65%).

The fixed appliance group showed a higher number of teeth with mild resorption (24 teeth, 95% CI: 16.32%–31.68%) than the clear aligner group (12 teeth, 95% CI: 5.82%–18.18%). The number of teeth with moderate or severe resorption was comparable between the 2 groups (fixed appliance group: 12 teeth with moderate resorption, 4 teeth with severe resorption; clear aligner group: 4 teeth with moderate resorption, 4 teeth with severe resorption).

The distribution of root resorption in upper anterior teeth differed significantly between the 2 groups (χ^2^ = 33.651, *P* < .001). The proportion of upper anterior teeth with moderate-to-severe resorption was significantly higher in the fixed appliance group than in the clear aligner group (40.91%, 72 teeth, 95% CI: 33.28%–48.54% vs 17.14%, 30 teeth, 95% CI: 11.56%–22.72%).

Additionally, the clear aligner group had more upper anterior teeth with no resorption (100 teeth, 95% CI: 92.65%–107.35%) than the fixed appliance group (68 teeth, 95% CI: 60.32%–75.68%). The number of upper anterior teeth with mild resorption was higher in the clear aligner group (44 teeth, 95% CI: 36.32%–51.68%) than in the fixed appliance group (28 teeth, 95% CI: 20.68%–35.32%).

No significant difference was found in the distribution of root resorption in lower anterior teeth between the 2 groups (χ^2^ = 6.746, *P* = .080). The fixed appliance group had more lower anterior teeth with no resorption (88 teeth, 95% CI: 80.32%–95.68%) than the clear aligner group (72 teeth, 95% CI: 64.32%–79.68%). The distribution of mild, moderate, and severe resorption was relatively balanced between the 2 groups (fixed appliance group: 40 teeth with mild resorption, 20 teeth with moderate resorption, 20 teeth with severe resorption; clear aligner group: 36 teeth with mild resorption, 24 teeth with moderate resorption, 36 teeth with severe resorption).

Detailed data on root resorption by tooth position are shown in Table 2.

**Table 2:** Root resorption by tooth position between the 2 treatment groups.

Tooth position	Resorption severity	Fixed appliances (n)	Clear aligners (n)	χ^2^	*P*	Fixed appliances 95% CI	Clear aligners 95% CI
Upper first molar	No resorption	20	28	11.333	.010	12.58%–27.42%	19.85%–36.15%
	Mild resorption	12	4	–	–	–	–
	Moderate resorption	12	4	–	–	–	–
	Severe resorption	12	20	–	–	5.82%–18.18%	12.58%–27.42%
Lower first molar	No resorption	16	36	15.692	.001	9.35%–22.65%	27.42%–44.58%
	Mild resorption	24	12	–	–	16.32%–31.68%	5.82%–18.18%
	Moderate resorption	12	4	–	–	–	–
	Severe resorption	4	4	–	–	–	–
Upper anterior teeth	No resorption	68	100	33.651	<.001	60.32%–75.68%	92.65%–107.35%
	Mild resorption	28	44	–	–	20.68%–35.32%	36.32%–51.68%
	Moderate resorption	36	12	–	–	–	–
	Severe resorption	36	12	–	–	–	–
Lower anterior teeth	No resorption	88	72	6.746	.080	80.32%–95.68%	64.32%–79.68%
	Mild resorption	40	36	–	–	–	–
	Moderate resorption	20	24	–	–	–	–
	Severe resorption	20	36	–	–	–	–

Only the vertical (*Y*-axis) movement of lower anterior teeth showed a statistically significant difference between the 2 groups (*P* = .017). The fixed appliance group had a mean vertical movement of 0.8814 ± 1.01909 mm (95% CI: 0.06107–1.82393 mm), while the clear aligner group had a mean vertical movement of 1.1314 ± 0.70874 mm (95% CI: 0.47595–1.78690 mm).

No significant differences were observed in the movement of upper anterior teeth (*X*-axis: *P* = .254; *Y*-axis: *P* = .722), upper posterior teeth (*X*-axis: *P* = .974; *Y*-axis: *P* = .140), or lower posterior teeth (*X*-axis: *P* = .283; *Y*-axis: *P* = .140) between the 2 groups (all *P* > .05). Detailed data on tooth movement are presented in Table 3.

**Table 3:** Comparison of tooth movement between the 2 appliance groups.

Tooth Movement Index	Fixed appliances (mm, Mean ± SD)	95% CI	Clear aligners (mm, Mean ± SD)	95% CI	*P*
Upper anterior teeth (*X*-axis)	1.3243 ± 0.81543	0.5701–2.0784	1.1679 ± 0.90268	0.3330–2.0027	.254
Upper anterior teeth (*Y*-axis)	1.3550 ± 1.34447	0.11158–2.59842	1.5643 ± 0.96898	0.66813–2.46045	.722
Upper posterior teeth (*X*-axis)	2.9043 ± 1.30929	1.6934–4.1152	2.4193 ± 1.73391	0.8157–4.0229	.974
Upper posterior teeth (*Y*-axis)	1.3036 ± 0.56197	0.78384–1.82330	2.4957 ± 1.76092	0.86714–4.12429	.140
Lower anterior teeth (*X*-axis)	1.0507 ± 0.29321	0.77954–1.32189	1.3633 ± 1.27657	0.23167–2.39976	.114
Lower anterior teeth (*Y*-axis)	0.8814 ± 1.01909	0.06107–1.82393	1.1314 ± 0.70874	0.47595–1.78690	.017
Lower posterior teeth (*X*-axis)	2.2350 ± 1.55823	0.79388–3.67612	1.7386 ± 0.64615	1.14098–2.33616	.283
Lower posterior teeth (*Y*-axis)	0.9993 ± 0.31776	0.70541–1.29316	1.3557 ± 1.46702	0.00106–2.71249	.140

*X*-axis: Horizontal movement; *Y*-axis: Vertical movement.

## 4. Discussion

### 4.1. Influence of orthodontic techniques on root resorption

Previous studies have shown that clear aligner therapy for Angle Class I malocclusion and anterior crowding cases has a root resorption incidence of 41.81% to 46%. Our results indicated a notably higher proportion of moderate-to-severe root resorption in the fixed appliance group (Table 1), which may be attributed to the use of maximum anchorage and continuous mechanical force in fixed orthodontics. From a biomechanical perspective, fixed appliances rely on archwire tension to drive tooth movement, and the continuous force applied during maximum anchorage retraction tends to concentrate stress at the root apex. This concentrated stress disrupts the balance of PDL metabolism, promoting excessive periodontal tissue remodeling and enhancing osteoclast activation – key biological processes underlying root resorption. In contrast, clear aligners deliver intermittent light forces through computer-simulated staged tooth movement. The intermittent nature of these forces creates intervals for PDL repair and remodeling, reducing the cumulative damage to root surfaces and thereby lowering the incidence of severe resorption.^[6]^ Notably, a higher proportion of severe resorption in the lower anterior teeth of the clear aligner group was significantly correlated with greater vertical movement (*P* = .017, Table 3). Biomechanically, vertical tooth movement involves complex interactions between the root and alveolar bone, as the root must traverse a larger volume of bone tissue compared to horizontal movement. This increased bone-root interaction may elevate mechanical stress on the PDL and root surface, suggesting that vertical movement may be an independent risk factor for root resorption. A recent study by further supported this finding, showing that the vertical movement distance of tooth roots is negatively correlated with the severity of root resorption.^[7]^

### 4.2. Tooth position-specific differences

#### 4.2.1. Anterior teeth

The fixed appliance group showed a significantly higher proportion of moderate-to-severe resorption in the upper anterior teeth (Table 2). Biomechanically, during maximum anchorage retraction with fixed appliances, the archwire exerts a posteriorly directed force on the upper anterior teeth, leading to concentrated apical pressure. This concentrated pressure can exceed the adaptive capacity of the PDL, triggering abnormal osteoclast activity and subsequent root resorption. Clinicians should be cautious of the potential risk of “apical contact with cortical bone due to insufficient torque control,” as improper torque management can further exacerbate apical stress concentration. For clear aligners, the higher proportion of severe resorption in the lower anterior teeth was directly related to greater vertical movement. Biomechanically, vertical displacement of lower anterior teeth requires overcoming resistance from the surrounding alveolar bone and PDL, and the increased mechanical load during this movement may induce more significant root surface damage. This indicates the need to optimize the treatment design in extraction cases – such as refining attachment placement or adjusting movement staging – to minimize unnecessary vertical movement and reduce associated resorption risks.

#### 4.2.2. Posterior teeth

The clear aligner group had a significantly higher proportion of no root resorption in the mandibular first molars (*P* = .001, Table 2). Biomechanically, clear aligners transmit force through the entire tooth surface via the tray, resulting in more dispersed force distribution across the root of posterior teeth. In contrast, fixed appliances apply force primarily through brackets and archwires, which may lead to localized stress concentration on posterior tooth roots. Additionally, clear aligners typically involve lighter posterior anchorage loading compared to fixed appliances, reducing the mechanical stress on mandibular first molars and lowering resorption risk. However, the clear aligner group showed a higher number of severe resorption cases in the upper first molars. This finding requires further validation with larger samples, as the small sample size limits the statistical robustness of this observation.^[4]^ Biomechanically, upper first molars in extraction cases may experience complex force interactions during space closure, and the current attachment design or force delivery pattern of clear aligners may not fully optimize stress distribution for these teeth, warranting further investigation.

### 4.3. Consistency and supplementary insights with previous studies

In non-extraction cases, the root resorption incidence of fixed orthodontics was reported to be 46% to 49.7%,^[6]^ which is consistent with the resorption pattern observed in our fixed appliance group. Fixed orthodontic appliances resulted in more root resorption compared to clear aligners, most notably in extraction cases.^[8]^ Related studies have also shown that patients treated with fixed orthodontic techniques exhibit more severe root resorption in the anterior teeth than in the posterior teeth,^[9,10]^ aligning with our finding of high moderate-to-severe resorption rates in upper anterior teeth with fixed appliances. Iglesias-Linares et al reported no statistically significant difference in the incidence of root resorption between fixed and clear aligners via logistic regression analysis,^[4]^ however, our study further revealed a higher risk of anterior resorption with fixed appliances in extraction cases – particularly for upper anterior teeth undergoing maximum anchorage retraction. This discrepancy may stem from the unique biomechanical challenges of extraction cases, such as the need for significant anterior retraction and space closure, which amplify the differences in force patterns between fixed and clear aligner systems. Additionally, the average treatment duration in both groups was long (37.93 ± 13.65 months), and previous research has identified treatment duration as an independent risk factor for root resorption.^[11]^ Prolonged exposure to orthodontic forces – regardless of appliance type – may accumulate PDL damage and root resorption, suggesting that long-term cases require enhanced imaging monitoring to detect early resorption.

### 4.4. Study limitations and future directions

This study has several limitations that should be acknowledged. First, as a single-center retrospective study, the sample size was relatively small – especially for molar subgroups – which may limit the generalizability of the results and the ability to detect subtle differences in resorption patterns. Second, root resorption was evaluated solely via panoramic radiographs, which have inherent limitations. Panoramic images are prone to up to 15% distortion in anterior and posterior regions, superimposition of adjacent structures, and inability to capture three-dimensional root morphology. These factors may lead to underestimation of subtle apical resorption or inaccurate assessment of resorption severity compared to three-dimensional imaging modalities such as cone-beam computed tomography (CBCT). However, CBCT was not used in this study due to its higher radiation dose especially for adolescent patients, who formed a large portion of the sample and limited availability in the study setting. To mitigate distortion, only radiographs with minimal positional errors assessed by a radiologist were included, and measurements were performed along the tooth’s long axis to align with the direction of least distortion. Third, the study did not account for potential confounding factors such as individual variations in bone density, PDL thickness, or genetic predispositions to root resorption, which may influence the observed results. Fourth, the biomechanical mechanisms underlying the observed resorption patterns – such as the specific stress distribution on different tooth roots during various movement types – were inferred based on existing literature rather than direct biomechanical measurements.

Future research should address these limitations by incorporating three-dimensional CBCT assessments to provide more accurate and detailed evaluations of root resorption.^[12]^ Larger multi-center studies with diverse patient populations are needed to validate the effects of tooth position and force patterns on resorption risk, particularly for molar subgroups. Additionally, future studies could integrate biomechanical modeling or in vitro experiments to directly measure stress distribution on root surfaces during fixed and clear aligner treatment, providing a more mechanistic understanding of resorption. Longitudinal studies with extended follow-up are also recommended to assess the long-term progression of root resorption after treatment completion. Furthermore, investigating the impact of individual factors on resorption susceptibility could help develop personalized treatment strategies to minimize resorption risk.

### 4.5. Clinical implications

The findings of this study have important clinical implications for orthodontic treatment planning in extraction cases. Clinicians should be aware that fixed appliances may be associated with a higher risk of moderate-to-severe root resorption in upper anterior teeth, particularly when maximum anchorage is used. To mitigate this risk, clinicians could consider using lighter forces, optimizing archwire sequences to reduce apical stress concentration, and enhancing torque control during anterior retraction. For clear aligner treatment, while the overall risk of severe resorption is lower, caution is warranted during vertical movement of lower anterior teeth. Clinicians should optimize treatment plans to minimize unnecessary vertical displacement – such as adjusting attachment design or refining movement staging – and closely monitor root resorption in cases involving significant vertical movement. Additionally, given the association between long treatment duration and resorption risk, clinicians should strive to shorten treatment time when feasible while maintaining treatment efficacy. Regular imaging monitoring – especially in long-term cases – can help detect early root resorption and allow for timely adjustments to treatment plans.^[13]^

It is important to emphasize that the results of this study indicate an association between orthodontic appliance type, tooth movement parameters, and root resorption risk, rather than a definitive causal relationship. The observed differences in resorption patterns may be influenced by multiple factors, including biomechanical force characteristics, tooth position, treatment duration, and individual patient traits. Therefore, clinicians should consider these findings alongside other clinical factors when selecting orthodontic appliances and designing treatment plans for extraction cases, balancing treatment efficacy with the goal of minimizing root resorption.

## 5. Conclusions

This retrospective study compared root resorption in 28 extraction patients with Angle Class I/II malocclusion treated with fixed appliances or clear aligners. Key findings: clear aligners were associated with a higher no-resorption rate and lower moderate-to-severe resorption in upper anterior teeth. Fixed appliances’ continuous heavy forces concentrated apical stress, while clear aligners’ intermittent light forces favored periodontal repair. Greater vertical movement of lower anterior teeth correlated with severe resorption in clear aligner treatment. Prolonged treatment also elevated risk, requiring enhanced imaging monitoring. Limitations include single-center design, small samples, and panoramic radiograph constraints. Clinically, optimize fixed appliance torque control and minimize clear aligner vertical movement. Future studies with 3D CBCT and larger cohorts are needed to validate findings.

## Acknowledgments

We sincerely appreciate the contribution of the participants to this study.

## Author contributions

**Conceptualization:** Li Su.

**Data curation:** Li Su.

**Formal analysis:** Li Su.

**Funding acquisition:** Li Su.

**Investigation:** Qichao Kang, Xu Zhang, Dan Li, Chen Luo, Zhe Tang.

**Methodology:** Jingwen Wang, Norma Ab Rahman.

**Resources:** Norma Ab Rahman.

**Validation:** Li Su.

**Visualization:** Li Su.

**Writing – original draft:** Li Su.

**Writing – review & editing:** Norma Ab Rahman.
